# Development of the systematic observation of COVID-19 mitigation (SOCOM): Assessing face covering and distancing in schools

**DOI:** 10.1017/cts.2021.786

**Published:** 2021-04-30

**Authors:** Ricky Camplain, Nanette V. Lopez, Dan M. Cooper, Thomas L. McKenzie, Kai Zheng, Shlomit Radom-Aizik

**Affiliations:** 1 Center for Health Equity Research, Northern Arizona University, Flagstaff, AZ, USA; 2 Department of Health Sciences, Northern Arizona University, Flagstaff, AZ, USA; 3 Institute for Clinical and Translational Science, University of California Irvine, School of Medicine, Irvine, CA, USA; 4 School of Exercise and Nutritional Sciences, San Diego State University, San Diego, CA, USA; 5 Department of Informatics, University of California, Irvine, Irvine, CA, USA; 6 Pediatric Exercise and Genomics Research Center, Department of Pediatrics, University of California Irvine, School of Medicine, Irvine, CA, USA

**Keywords:** SARS-CoV-2, pediatric, school, direct observation, prevention

## Abstract

**Introduction:**

During the COVID-19 pandemic, some K-12 schools resumed in-person classes with varying degrees of mitigation plans in the fall 2020. Physical distancing and face coverings can minimize SARS-CoV-2 spread, the virus that causes COVID-19. However, no research has focused on adherence to mitigation strategies during school days. Thus, we sought to develop a systematic observation protocol to capture COVID-19 mitigation strategy adherence in school environments: The Systematic Observation of COVID-19 Mitigation (SOCOM).

**Methods:**

We extended previously validated and internationally used tools to develop the SOCOM training and implementation protocols to assess physical-distancing and face-covering behaviors. SOCOM was tested in diverse indoor and outdoor settings (classrooms, lunchrooms, physical education [PE], and recess) among diverse schools (elementary, secondary, and special needs).

**Results:**

For the unique metrics of physical-distancing and face-covering behaviors, areas with less activity and a maximum of 10–15 students were more favorable for accurately capturing data. Overall proportion of agreement was high for physical distancing (90.9%), face covering (88.6%), activity type (89.2%), and physical activity level (87.9%). Agreement was lowest during active recess, PE, and observation areas with ≥20 students.

**Conclusions:**

Millions of children throughout the USA are likely to return to school in the months ahead. SOCOM is a relatively inexpensive research tool that can be implemented by schools to determine mitigation strategy adherence and to assess protocols that allow students return to school safely and slow the spread of COVID-19.

## Introduction

The impact of the coronavirus disease 2019 (COVID-19), caused by the novel coronavirus, severe acute respiratory syndrome coronavirus 2 (SARS-Cov-2), has been widespread, with 30 million cases diagnosed and over 550,000 deaths in the USA as of March 31, 2021. When COVID-19 was declared a pandemic, schools were among the first operations to close to prevent community spread. However, in the fall of 2020, 56 million school-aged children (5–17 years of age) resumed education as some schools opened to in-person classes with varying public health mitigation plans [1]. Children and adolescents comprise asymptomatic, symptomatic, and hospitalized populations of SARS-CoV-2-infected individuals and their capability of spreading disease to school staff and family has yet to be resolved [2–5]. However, mitigation procedures can minimize SARS-CoV-2 outbreaks even in the close quarters of overnight summer camps [6].

Viral aerosolization can occur due to the general social and physically interactive nature of school-aged children during classroom learning, communal dining, recess, and physical education (PE) classes. Comprehensive return-to-school plans emphasize adherence to accepted SARS-CoV-2 viral mitigation strategies procedures, including physical distancing (staying at least 6 feet from others who are not from the same household) and face cover wearing [7–9]. Specifically, new guidelines released by the Centers for Disease Control and Prevention highlight the need to promote behaviors that reduce COVID-19’s spread, including maintaining healthy environments and operations, and preparing for when someone gets sick [10]. Recognizing mitigation strategies represent unnatural behaviors for K-12 students, attention has been focused on strategies to implement and operationalize mitigation protocols in schools. Little emphasis has been given to how to quantify adherence. Without adherence metrics, effectiveness of SARS-CoV-2 transmission mitigation procedures cannot be ascertained.

Existing instruments for quantifying health behaviors could be applied to assess the fidelity of COVID-19 mitigation. These include self-report [11], wearable technologies [12–17], and direct observation by trained personnel [18–21]. Self-reports of daily living (e.g., physical activity (PA) or diet) by children and adolescents are highly inaccurate [18–21]. Wearable technologies (accelerometers) are increasingly used to gauge PA and sedentary behavior in children [22,23], but necessary technological advances are not designed to assess physical-distancing or face-covering behaviors [24–26]. Moreover, the use of cell phone- or GPS-monitoring in school-aged children and adolescents raises questions of health data privacy [27], embodied in FERPA and HIPAA regulations.

Direct observation approaches developed to assess levels of PA in a variety of real-world environments were readily adapted to measure COVID-19 mitigation strategies in K-12 schools. The original System for Observing Play and Leisure Activity in Youth (SOPLAY), designed for PA settings in schools, and the System for Observing Play and Recreation in Communities (SOPARC), designed to include playgrounds and parks, were designed to obtain direct information on PA in open spaces [20,28]. SOPLAY and SOPARC are based on momentary time sampling techniques in which systematic and periodic scans of individuals and contextual factors in PA environments are made and they have been adapted for multiple settings [21]. Given SOPARC’s proven ability to obtain reliable observational data, we sought to test a new strategy of using systematic observation to capture COVID-19 mitigation strategy adherence, including physical distancing and face coverings, among grade-school-aged children in diverse school settings: The Systematic Observation of COVID-19 Mitigation (SOCOM).

## Materials and Methods

### Study Setting

Four schools in Orange County, California, were recruited to participate in a Safe School Restart study to begin essential research on COVID-19 transmission in children and adolescents. The aim of Safe School Restart was to determine how effectively state and regional guidelines slowed viral transmission as schools restarted. Private schools across the country have been able to expend the resources necessary to develop and implement effective mitigation strategies. Public schools, particularly Title 1 public schools, have had greater difficulty in accessing necessary resources [29,30]. Consequently, our study includes four representative schools in Orange County, California:

1. Private K-12 school serving predominantly middle- and upper-middle socioeconomic status (SES) students.
2. K-6 public school serving predominantly Latino, lower SES students.
3. K-8 public charter school located in a predominantly Latino, lower SES neighborhood.
4. K-6 public charter school serving predominantly children with special needs, including down syndrome, autism spectrum disorders, etc.

The participating schools had varying levels of COVID-19 plans that were made in effort to meet the CDC guidelines that were in place during the study period. Plans included thoughtful and thorough screening, mask wearing, social distancing, hand hygiene, guest visitations, and testing procedures. All schools had some level of in-person learning with three schools permitting only a small proportion of students to attend in-person.

SOCOM was designed to observe during four distinct school day sessions: classroom learning, active recess (regularly scheduled periods for PA and play that was monitored by trained staff or volunteers), instructional PE classes, and communal dining (lunch).

### Partnerships with Schools

Schools were selected for Safe School Restart to reflect the wide range of SES, ethnic diversity, and school layouts and facilities that exist within Orange County, California, allowing for a determination of the viability of the SOCOM tool in different settings (Table 1). Building on previous relationships, the schools were approached as colleagues and partners, an approach that facilitated approvals for the study by schools and school districts. Great care was taken to plan with school staff, so elements of the study were clear, acceptable, and followed current school policies established in response to the COVID-19 pandemic.

**Table 1.** Characteristics within observed schools

Observation Area	Description	Number of students
**Private K-12 school (fifth grade)**		
Classrooms	Scenario 1: Students had their own desks at least 6 ft from others. NOTE: Occasionally students moved their desks and chairs closer to others and were not 6ft apart). Scenario 2: Students shared a desk and had a plastic divider between them.	16–18
Communal dining	There were 36 rectangle tables, 6 circle tables, and 6 square tables. Rectangular tables had designated stickers to identify where students could sit. Circle and square tables had plastic dividers on the tabletop. Custodians cleaned the tables during transition times from one class to another.	∼4–6 at each table
Active recess	Multiple areas were used for active recess that were shared with other grades. A rotation schedule for shared areas was created.	∼50
Physical activity/physical education class	Two outdoor grass fields, basketball courts, tennis courts, and an indoor gymnasium area were available for physical activity/physical education classes.	18
**Private K-12 school (middle school)**		
Classroom learning	Scenario 1: Lecture classrooms had one desk per student. Scenario 2: Laboratory classrooms had students share benches in which students sat 6 ft apart with or without dividers.	14–16
Communal dining	There were 36 rectangle tables, 6 circle tables, and 6 square tables. Rectangular tables had designated stickers used to identify where students could sit. Circle and square tables had plastic dividers on the tabletop. Custodians cleaned the tables during transition times from one class to another. Most students sat together in large numbers.	≥4 at tables, lounge areas, or grass
Active recess	No recess available.	0
Physical activity/physical education class	Two outdoor grass fields, basketball courts, tennis courts and an indoor gymnasium area were available for physical activity/physical education classes.	∼18
**K-6 public school**		
Classroom learning	Students had their own desks placed 6 ft apart.	14
Communal dining	There were rectangle lunch tables that had two students per table sitting on opposite sides.	14
Active recess	Students could use a basketball court.	8–10
Physical activity/physical education class	One grass location was available for physical activity/physical education classes.	14
**K-8 public charter school**		
Classroom learning	Students had their own desks with a plastic U-shaped divider in one classroom.	12
Communal dining	Lunch was provided in the classroom and students ate at their own desk.	12
Active recess	No recess was available.	0
Physical activity/physical education class	No physical activity/physical education classes were available.	0
**K-6 special needs public charter school**		
Classroom learning	Students sat at their own desk placed 6 ft apart with plastic U-shaped dividers in one large classroom (30 × 15 feet).	10
Communal dining	Students ate lunch at tables in a room (150 × 75 feet) in which siblings could sit close to one another while other students were on the opposite end of the lunch table from other students. In many cases, students did not sit 6 ft apart.	10
Active recess	A large grass area and basketball court (150 × 75 feet) were available	10
Physical activity/physical education classes	Classes were provided using online instruction (ZUMBA) inside a classroom.	10

### SOCOM Development

**Fig. 1.**
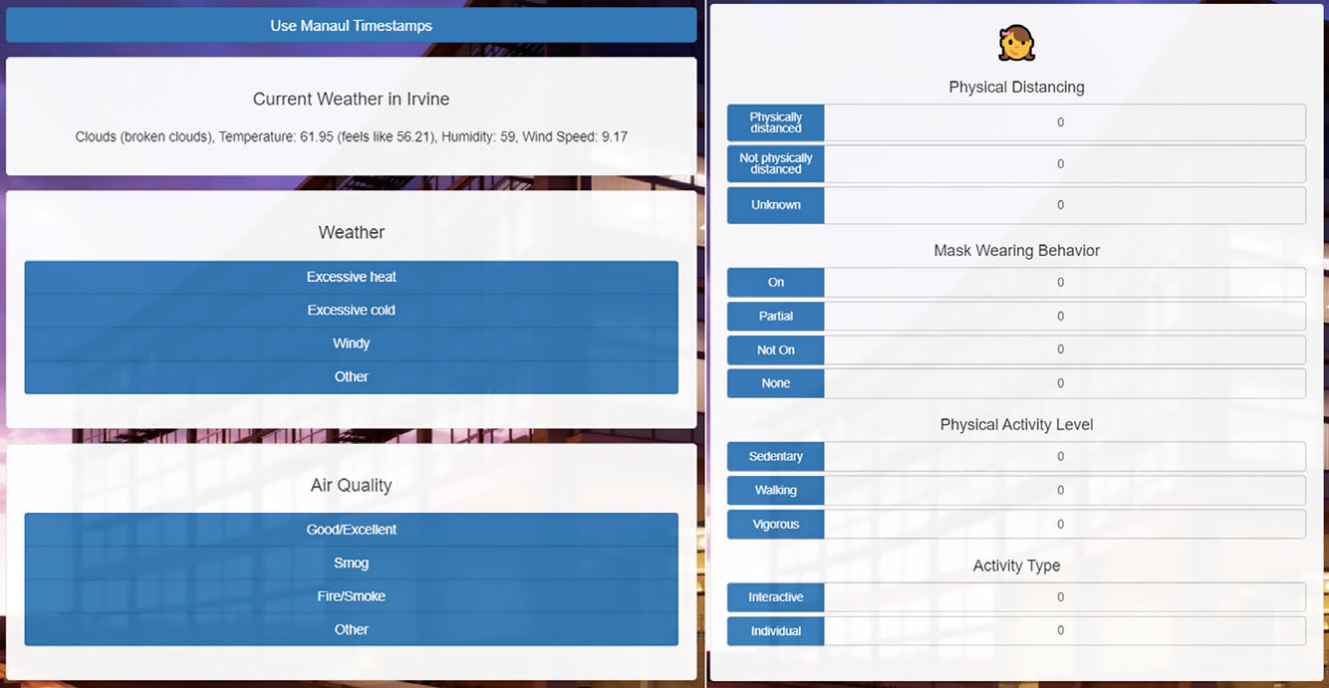
Sample Systematic Observation of COVID-19 Mitigation (SOCOM) Electronic Data Collection Form.

**Fig. 2.**
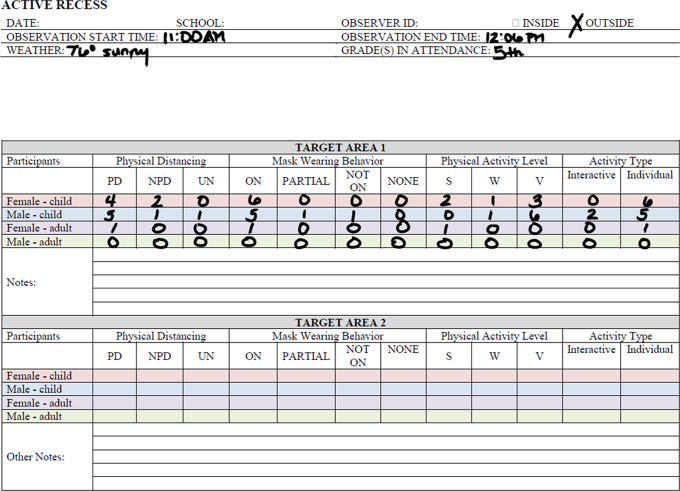
Sample Systematic Observation of COVID-19 Mitigation (SOCOM) Paper Data Collection Form.

In the summer of 2020, the SOCOM protocol was developed to focus on assessing COVID-19 mitigation procedures (physical-distancing and face-covering behaviors). Paper and electronic observation forms were developed to capture data (Figs. 1 and 2). The custom electronic data capture system optimized for mobile devices was developed by the Center for Biomedical Informatics, UC Irvine Institute for Clinical and Translational Science. The platform leverages the Bootstrap responsive user interface framework and was programmed in ASP.Net C#. In addition to the SOPARC-based data fields that research coordinators can enter manually based on their field observations, the system is also capable of obtaining local weather information automatically through an application programming interface (API) provided by openweathermap.org. All data captured are stored in a HIPAA-compatible environment hosted at the Enterprise Data Center of the UCI Health.

### Measures

Primary measures included physical-distancing, face-covering behavior, PA levels, and activity type (Table 2). In addition, observed individuals were categorized into sex/age groups (female students, male students, female adults, and male adults). Data were recorded to identify specific observation target area, date, grades in attendance, time of observation, and weather conditions.

**Table 2.** Systematic observation of COVID-19 mitigation (SOCOM) central measures

Variable	Definition
Physical distancing	1. *Physically distanced* – observed individual was not within six feet of another person 2. *Not physically distanced* – individual being observed was within six feet of others 3. *Unknown* – observer could not determine if observed individual was within six feet of others
Face covering behavior	1. *On* – individual had both his/her nose and mouth fully covered 2. *Partial* – individual had face covering on but either his/her nose or mouth was not covered 3. *Not on* – individual had no face covering on, but one was visible (e.g., mask in hand or hanging from ear) 4. *None* – individual had no visible face covering anywhere on body
Physical activity levels	1. *Sedentary* – individual was lying down, sitting, or standing in place 2. *Walking* – individual was engaged in walking at normal pace 3. *Vigorous* – individual was engaged in an activity more vigorous than an ordinary walk (e.g., causing increased heart rate, such as when jogging, swinging, doing cartwheels).
Activity type	1. *Interactive* – individual was engaged in an interactive activity where he/she was using a shared piece of equipment or touched another person 2. *Individual* – individual was engaged in an independent activity with no shared equipment

### Training

In preparation for SOCOM, observers (research assistants from the University of California, Irvine’s Pediatric Exercise and Genomics Research Center) studied the written protocol and participated in a 4-hour workshop. Original written and video SOPARC training materials were used during the workshop (https://activelivingresearch.org/soparc-system-observing-play-and-recreation-communities). Training included reviewing definitions and coding conventions, differentiating among codes, coding practice, and orientation to the observation tools. Observer preparation also included target area mapping strategies. Coding conventions and how to differentiate among various PA levels included video lectures and practice with feedback from videotaped samples through original SOPARC training. Distinguishing between face coverings, physical distancing, and activity type definitions was taught using photographs and videos. Observers practiced coding and received feedback on their scoring before engaging in field practice.

### Observation Preparation

Maps of school grounds from Google Maps or school administrators were used to precisely identify outdoor target areas, spaces in which activities might occur and were small enough to accurately code people using them. The size, location, and boundaries for both indoor and outdoor target areas were identified and the sequential order for observing them established.

As COVID-19 recommendations and restrictions varied among schools and time periods, visiting them was imperative before finalizing school-specific observation protocols and visitation days. Administrative-level school officials escorted observers around the school to note the general layout and major features of the facilities and to specifically identify potential target areas for observation.

Before official data collection began, all observers attended a field-based training at one of the schools using the developed protocol and data collection form to practice using the protocol and form. Observers used the schedule of observations like that for official observations but were able to discuss protocols and category classifications.

### Recording Procedures

To establish reliability during visits to target areas, two to three observers simultaneously completed observations either by using paper forms or individual cell phones (Figs. 1 and 2). Timestamps and weather information were uploaded automatically into the electronic form.

After recording the characteristics of a target area, the observers simultaneously completed area scans by making independent “visual sweeps” from left to right using the same pace. Separate scans were conducted for female students, male students, female adults, and male adults, and each characteristic (physical distancing, face covering, PA, and activity type) for a maximum of 16 scans per target area. For example, during the first scan for female students, observers recorded whether each was physically distanced, not physically distanced, or unknown. During the second scan, observers recorded data for each female student’s face covering. For PE classes and active recesses, a third scan was performed to record the PA level of each female student as sedentary, walking, or vigorous. Finally, for female students, a scan was made to categorize each as being interactive or individual. This scanning procedure was then completed for male students, female adults, and male adults in the target area (if any). Observers then moved to the next target area. Occasionally (e.g., unusual large numbers of people in the area), target areas were subdivided into smaller sub-areas so more accurate measures could be obtained and data for these sub-areas were later summed to provide an overall measure. During groups of scans, observers also took qualitative notes on contextual information. When needed, observers used paper observation forms and later entered the information into the electronic data format.

Weekly meetings were conducted with observers and research team to discuss challenges experienced during observations and to review the study protocol.

### Planned Data Analysis

For physical-distancing, face-covering behavior, PA level, and activity type, counts will be tallied for those engaged in each group in each school and observation area to obtain a summary score for female students, male students, female adults, and male adults. A proportion of individuals in each physical-distancing scenario, face-covering behavior, PA level, and activity type will be calculated by age/sex group.

## Results

### Observations

During a school visit, observation times varied by the number of target areas and people present and ranged from 2 min (in classrooms) to 15 min (in dining rooms). Communal areas generally had more people and more target areas and more scans were required. When large numbers of people were present (>20), some observers preferred the paper entry format compared to the electronic form as they could code more easily. Thus, areas with less activity and target areas with a maximum of 10–15 students were more favorable for accurately and confidently capturing data.

The presence of observers may have influenced staff and student behavior, especially indoors. Schools had restrictions on visitors due to COVID-19 protocols and teachers sometimes inquired why observers were there. Meanwhile, when students were more physically active (e.g., PE classes) observers typically went unnoticed.

### Reliability

Reliability data for PA, physical-distancing, and face-covering behaviors were collected during all observation periods using two assessors who made simultaneous, independent observations. The overall proportion of agreement from 166 scans was calculated for each variable (face covering, 88.6%; physical distancing, 90.9%; and activity type, 89.2%). When assessed by observation area, agreement was lowest during active recess and PE classes (79.7% to 99.1%). Agreement was lower when there were ≥20 students present (80.2%) compared to <20 students (90.2%). Reliability for PA level calculated for 34 observations (collected during active recess and PE classes) showed the proportion of agreement was 87.9%, similar to previous reliability studies using SOPARC [28].

### Preliminary Results

The SOCOM activity codes had been used in other observation systems [20,31,32], and with their construct validity being established via heart rate monitoring among 4- to 18-years-old children [31,33] and with accelerometers in schools [34]. Subsequently, we were able to compare PA results to previous studies.

During recess in the three schools that provided recess, 25.8% of students were sedentary, 21.5% walked, and 52.8% engaged in vigorous activity. The proportion of students engaged in walking or vigorous activity (74.2%) was higher than those in previous studies (51–68%) [20]. Overall during PE classes, 38.6% of students were sedentary, 25.0% were walking, and 36.4% were engaged in vigorous activity. Meanwhile, the PE class program differed substantially by school as did the resulting activity levels. Students in the K-6 special needs public charter school had PE within their classrooms and subsequently a high proportion of them were sedentary (87.5%) as students were still in the classroom. In the private and public schools providing formal PE classes, 34% of students were sedentary.

Additional preliminary data on mitigation using SOCOM linked to SARS-CoV-2 infection in the four schools are also available [35].

## Discussion

Systematic observation has been widely used in collecting information on children’s and adolescents’ behaviors [20,21,28,31]. Therefore, we sought to test a new strategy of using systematic observation to capture COVID-19 mitigation strategy adherence, including physical distancing and face coverings, among grade-school-aged children in the school environment. SOCOM may serve as a reliable and useful tool in assessing COVID-19 mitigation procedures in schools.

To safely plan for in-person learning, K-12 schools must understand mitigation strategy adherence among their students and staff. Because physical-distancing and face-covering behaviors are two recognized methods to prevent the spread of COVID-19 [36], real-time response to poor adherence is an important component of safely re-opening and keeping K-12 schools open for in-person learning.

Following data collection, a verbal summary of the results was provided to each school and formal presentations of the findings and additional COVD-19 findings from the Safe School Restart Study are in progress. SOCOM can be applied by schools to monitor compliance, adjust mitigation strategy messaging, and contribute to informed school policies. The current study was limited to observing only traditional classrooms, communal dining, active recess, and PE classes. Nonetheless, we believe the tool can be used in other school settings, such as music classes, laboratories, teachers’ lounges, and hallways as well as when students arrive at and depart the school grounds. Additional research, however, is needed to assess the viability of using SOCOM in these areas and times.

Interrater reliability profiles for SOCOM were comparable to those found with SOPLAY and SOPARC [28]. After observations, the research team discussed the effectiveness of the SOCOM protocol, observation form, and experience. In general, heuristic assessments of SOCOM was positive with certain limitations in using the electronic form, including the need for access to the internet on the devices used. Although there were limitations with concurrent electronic data entry, observers had access to the paper data collection forms and were able to later enter data into the electronic form. SOCOM worked well, even within the constraints of school environments during the pandemic. However, our study did not train school personnel as observers.

Although we have not yet trained in-school personnel to collect data, we believe the high reliability, ease of training and use, and the ability to modify the instrument, SOCOM could be used by school administrators and staff to help assess school reopening. Interventions (e.g., policies) could subsequently be implemented to further protect students, teachers, families, and the local community.

Due to the complex, novel nature of capturing COVID-19 mitigation strategy adherence, training and observation preparation were important. Training materials for SOCOM were obtained from existing valid and reliable methods. Although previous training materials focused mainly on capturing PA levels, we found that training to observe physical-distancing and face-covering behaviors was readily adaptable. Field-based observational training using the protocol in real-time were imperative. Our training included an initial visit to each school to familiarize observers with features of the school’s physical environment and review details of the school’s COVID-19 schedule, rules, and restrictions. Visits permitted the development of appropriate weekly and daily observation schedules for each school and was welcomed by school leadership and staff. Visits were necessary for positioning observers in areas where they could have a clear view and to minimize interactions with students and staff. However, a limitation of SOCOM is the potential for observation bias as individuals may be inclined to be on their best behavior while under observation. To decrease observation bias in the future, observers should be school personnel. Training teachers, staff, and administration to use SOCOM in schools can also provide schools with real-time feedback that may impact practices and policies for in-person learning.

## Conclusions

Despite fears of possible surges of COVID-19 cases due to SARS-CoV-2 variants, millions of children and staff will be in schools under various conditions. Moreover, COVID-19 the vaccination of adults in the USA is proceeding, the vaccination of enough people to approach herd immunity has yet to be achieved. At the time of this writing, a vaccine has not been approved to be used among children <16 years old and no child under the age of 12 years has been enrolled in any safety or efficacy vaccine trials, thus the potential for widespread COVID-19 vaccinations in school-aged children to diminish the need for mitigation procedures in schools is many months away. Quantifying the success of SARS-CoV-2 transmission mitigation in school settings is likely to be useful for the foreseeable future. Standardized methods of measuring the fidelity of mitigation procedures will likely aid in identifying the most effective ways to minimize SARS-CoV-2 viral transmission. SOCOM is a relatively inexpensive tool that can be implemented by schools in various settings, including the school day, after school programs, and school sports and competitions, to determine mitigation strategy adherence to help students return to school safely and slow the spread of COVID-19.
